# Ratiometric multisensing with heteroaggregates of aqueous carbon quantum dots and rare earth doped nanocrystals

**DOI:** 10.1039/d6na00343e

**Published:** 2026-07-13

**Authors:** Albenc Nexha, Tobias Kraus

**Affiliations:** a INM-Leibniz Institute for New Materials Campus D2 2 66123 Saarbrücken Germany tobias.kraus@leibniz-inm.de; b Saarland University, Colloid and Interface Chemistry 66123 Saarbrücken Germany

## Abstract

Combinations of two different colloidal nanostructures can inherit properties of both and give access to new, emerging properties. Here, we create optically downconverting heteroaggregates of carbon quantum dots and lanthanide doped nanocrystals with high quantum yields and stability against blinking and bleaching. These heteroaggregates are heavy metal-free and display visible emissions that are suitable for multimodal sensing. Red emitting europium Eu^3+^ doped CaF_2_ nanoparticles were combined with orange emitting carbon quantum dots prepared by solvothermal reactions. Simple mixing led to the formation of heteroaggregates as confirmed by dynamic light scattering and zeta potential analysis. The assembly caused electromagnetic coupling, changed the particles' response to temperature and pH in aqueous dispersion, and increased the sensing performance. Emission dropped by 39% at 550 nm (of carbon quantum dots) and 8% at 613 nm (of Eu^3+^ doped CaF_2_ nanocrystals) from 17% and 33% of the pure nanoparticle dispersions. Changing pH from 1.3 to 12.5 quenched emissions by 92% and 74% at the same wavelengths, increasing from 62% and 47% of the pure dispersions. Ratiometric calibrations based on the intensity ratio of the 550 nm and 613 nm emissions were constructed to use the heteroaggregates as sensors. They performed as optical thermometers in the range from 293 K to 333 K with a temperature resolution of 0.35 K, and pH values within a range from 1.3 to 12.5 with a resolution of 0.15 pH units. Our results show that heteroaggregates of coupled nanostructures provide improved performance in temperature and pH sensing.

## Abstract 2

Förster resonance energy transfer heteroaggregates of carbon quantum dots and europium doped nanocrystals can ratiometrically readout temperature and pH in aqueous media.

## Graphical abstract

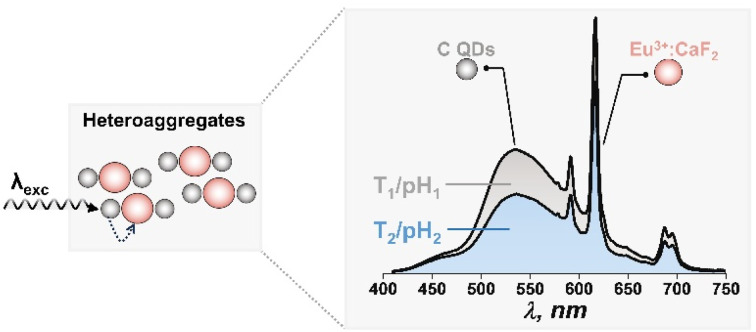

## Introduction

Photoluminescent nanomaterials absorb and emit light in spectral ranges that can be tuned *via* size, shape, and composition.^1^ They are used in applications ranging from bioimaging and medical diagnostics to LEDs, displays and environmental sensing.^1^ Environmental sensing with such nanomaterials relies on their spectral response to temperature, relative humidity, or pollutants that cause measurable changes in the intensity, wavelength or lifetime of their photoluminescence.^2–4^ A large surface-to-volume ratio leads to rapid responses, improves sensitivities and enables high temporal resolutions.^3,5^ Strong optical responses are possible that are visible even to the naked eye.^3,5^ These properties have been exploited for remote, real-time and non-invasive monitoring of environmental parameters.^3,5^ Sensors based on such materials have been used to create self-deployable and biodegradable soft robots for monitoring environmental parameters in air and topsoil.^6–8^

Carbon quantum dots (C QDs) are photoluminescent nanomaterials that were discovered during the purification of single walled carbon nanotubes,^9^ and have been used in the optical sensing of pesticides, antibiotics, heavy metals, and environmental parameters.^2,3^ C QDs can be prepared using straightforward green chemistry protocols using low-cost abundant precursors.^3^ They display broadband photoluminescence with high quantum yields (typically photoluminescence quantum yields PLQY >60%),^3^ have remarkable photostability and do not exhibit blinking like semiconductor quantum dots.^2^ Biomedical sensing applications show their practical applicability in medicine.^2,3^

Most published sensor applications with C QDs rely on a single wavelength emission with an intensity that either quenches “off” or “on” as a function of the environmental parameter or analyte being readout.^2,3,10^ For example, heavy metal ions such as Hg^2+^ quench “off” after adsorption due to the strong affinity of the metal for the carboxylic groups on the surface of C QDs.^11^ Temperature affects the emission by the thermal activation of non-radiative decay pathways.^12^ It is possible to use this change for chemosensing or thermometry, but single-wavelength readout is inherently sensitive to the C QD concentration, fluctuations in the excitation source and environmental factors, which limits accuracy and sensitivity.^2,3,10^ Recently, dual emissive C QDs have been used to detect pH in solution or at intercellular levels,^12–15^ and temperature.^12^ In a typical experiment, C QDs were excited with a 365 nm laser to trigger emissions at 393 nm and 580 nm, whose ratio changed linearly with pH.^13^ It is often difficult in a single C QD spectrum to find emissions for ratiometric readout that do not overlap,^14^ and can be clearly separated.^12^ This limits the precision of the ratiometric sensors based on such particles.^4,16^

Lanthanide doped nanocrystals (Ln NCs) display upconverting, downconverting or downshifting emissions, depending on their composition.^4,16,17^ In contrast to C QDs, Ln NCs have sharp and well-defined emission maxima with long luminescence lifetimes (microsecond to millisecond range,^4,18^ compared to the nanosecond range for C QDs).^3^ The 4f electronic transitions of lanthanide ions are protected by outer 5s and 5p orbitals, rendering the optical properties unaffected by the host's lattice and environmental fluctuations. Typically, Ln NCs, such as Er^3+^, Tm^3+^, Eu^3+^ or Tb^3+^, display several electronic transitions under a single excitation.^4,16^ The emitters do respond to temperature (from cryogenic to approximately 1200 K),^4,8,16^ and pH (from 4 to 11),^19,20^ but the quenching is limited. Ln NCs based on upconverting structures have been used as thermal and relative humidity probes.^2,3,8,21^ The intensity ratio of the green and red emissions of Er^3+^ ions was used to determine the temperature of topsoil,^6^ or the relative humidity in air,^7^ with intensity changes below 30%.^6^

We are interested in heteroaggregates of C QDs and Ln NCs that combine the distinct photoluminescence profiles and quenching characteristics of the two materials. Our goal is to enable a ratiometric readout of the quenching with a high signal-to-noise ratio (SNR).^2,3^ We aim at heteroaggregates where one photoluminescent nanomaterial acts as a reference (*e.g.*, Ln NCs with nearly constant emission) and the other is actively quenched (*e.g.*, C QDs). Conjugating them ensures that they are in close spatial proximity so that the emission is homogeneous and not angle-dependent. In addition, we evaluate whether electromagnetic coupling between the particles occurs and how it affects the SNR. Our heteroaggregates differ from existing combinations of C QDs and downconverting microparticles^22^ in optical scattering and coupling strength.

In the following, we create heteroaggregates from C QDs and europium doped nanoparticles (Eu^3+^:CaF_2_), prepared using solvothermal protocols. C QDs show bright orange emission at 550 nm, while Eu^3+^:CaF_2_ has the characteristic red emission of Eu^3+^ at 613 nm. Both particle types are dispersible in water and mixing leads to the formation of heteroaggregates. We analyse the photoluminescence of the heteroaggregates upon 395 nm UV excitation as a function of particle number ratio, temperature, and pH. The intensity ratio of the 550 nm and 613 nm emissions in aqueous dispersion is used to analyse the sensitivity, temporal response, reversibility and accuracy for detection of temperature (293 K to 333 K) and pH (1.3 to 12.7).

## Experiments

### Materials

All chemicals were purchased from Sigma-Aldrich and used without further purification.

### Synthesis of carbon quantum dots

A solvothermal route yielded water soluble carbon quantum dots.^10^ Briefly, a mixture of 0.04 g 2,7-naphthalenediol and 0.02 g urea was dissolved in 10 mL of ethanol under ultrasound for 5 minutes and transferred to a 50 mL Teflon lined autoclave. Particles formed at 200 °C for 12 h. After cooling down to room temperature, the quantum dots were filtered with a microporous membrane (0.22 µm). The solvent was removed using a rotary evaporator and dialyzed against a dialysis bag (MW = 3500 Da) for 7 days. Carbon quantum dot powders were obtained after further drying at 80 °C in vacuum.

### Synthesis of europium doped CaF_2_ nanocrystals

A solvothermal methodology was applied to yield water soluble europium doped CaF_2_ nanocrystals.^23^ In short, 1.75 mmol of the metal acetates (5 mol% europium acetate and 95 mol% calcium acetate) were dissolved in 5 mL distilled water for 30 minutes. 5 mL of aqueous solution of sodium citrate (10 mmol) was added, and the mixture was stirred overnight, followed by the addition of a 3.5 mL aqueous solution of 4.375 mmol of NH_4_F. After stirring for 30 minutes, the mixture was transferred to a 50 mL autoclave reactor and particles formed at 180 °C for 6 hours. After centrifugation at 8000 rpm for 10 min, followed by washing with ethanol and distilled water, citrate coated nanocrystals dispersed in water were obtained as the final product.

### Characterization of the fluorescent materials

Transmission electron microscopy (TEM) images were acquired with a JOEL JEM 2100 machine operating at an acceleration voltage of 100 kV. TEM specimens were prepared by depositing around 15 µL of a diluted dispersion of the materials on the surface of a carbon-coated copper grid. Selected area electron diffraction patterns were acquired under high magnification TEM. The sizes of 100 nanoparticles were determined using ImageJ. The crystalline structures were characterized by X-ray powder diffraction (XRPD) using a D8 Advance diffractometer with a copper source CuKα radiation (*λ* = 1.54060 Å, 40 kV, 40 mA) with a 2*θ* range from 10 to 80° and a scan rate of 0.02° per second. The phonon energies of the materials were characterized by Raman spectroscopy. A Renishaw inVia microscope with unpolarized light from a 532 nm argon laser was focused on the dried materials using a 50× objective. Analysis was performed within the range of 100–2000 cm^−1^, using a grating with 2400 lines per mm and an exposure time of 10 s. Digital images of the fluorescent dispersions were acquired using the camera of an iPhone 14 Pro (48-megapixel quad-pixel sensor). The average hydrodynamic diameter of the nanoparticles was determined from Dynamic Light Scattering (DLS) using an Anton Paar Litesizer 500 using a 658 nm laser wavelength.

### Sensing temperature and pH with the heteroaggregates of carbon quantum dots and europium doped nanocrystals

Heteroaggregates were prepared by mixing different concentrations of equal amounts of aqueous solutions of the carbon quantum dots and the europium doped nanocrystals at room temperature under stirring, followed by 5 min of ultrasound. The zeta potentials of the carbon quantum dots, europium doped nanoparticles and heteroaggregates were measured *via* electrophoresis in a Nano ZS90 (Malvern, USA) Zetasizer. The photoluminescence spectra of the particles and heteroaggregates were analysed *via* a commercial spectrofluorometer FS5 from Edinburgh Instruments, equipped with a built-in xenon laser operating from UV to NIR wavelengths. The laser is oriented perpendicular to the position of the nanoparticles, which are placed within a quartz cuvette inserted into a SC-25 temperature-controlled module. The emission spectra were acquired within the range from 400 nm to 700 nm with a resolution of 5 nm and integration time of 2 s. The quantum yield of the photoluminescence generated from the carbon quantum dots and europium doped nanocrystals was determined using the absolute method *via* an integrated sphere linked to the spectrofluorometer. The photostability of the fluorescent materials was acquired under these conditions during a period of 24 hours.

The temperature dependence of the photoluminescence was monitored within the range from 293 K to 333 K using the SC 25 module with a ramp of 20 K min^−1^ and an accuracy of ±0.1 K. Repeatability was ensured by recording the photoluminescence of the heteroaggregates under 5 cycles of heating (to 333 K) and cooling (to 293 K). The pH dependence of the photoluminescence was studied in a phosphate buffer saline (a 10 mM PBS containing 150 mM NaCl) at various pHs, adjusted by adding adequate concentrations of HCl and NaOH. Approximately 0.2 mL of the respective buffer was added to aqueous dispersions of the heteroaggregates and photoluminescence data were recorded simultaneously.

## Results and discussion

**Fig. 1:**
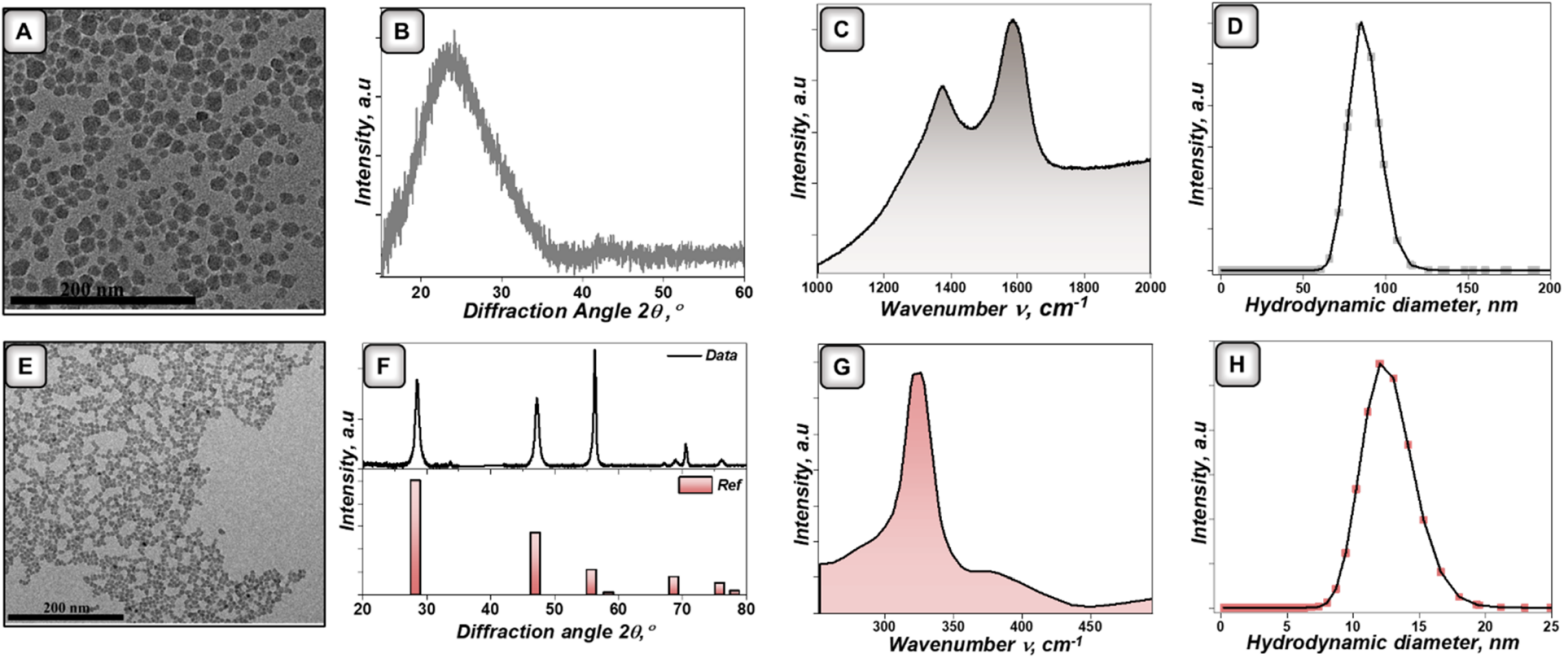
Structures of the pure particles: transmission electron microscopy (TEM) images, X-ray diffraction patterns, unpolarized Raman spectroscopy, and dynamic light scattering hydrodynamic diameter of: (A)–(D) carbon quantum dots and (E)–(H) Eu^3+^ doped CaF_2_ nanoparticles produced *via* solvothermal routes.

Water soluble C QDs were prepared *via* a solvothermal reaction.^10^ The quantum dots were bimodal with a quasi-spherical shape with average diameters of 5 ± 0.7 nm (Fig. 1A). Selected area electron diffraction (SAED) revealed lattice fringes with a crystal plane spacing of 0.21 nm,^10^ indicating a (100) graphite plane for the C QDs (Fig. S1, SI). A single XRD peak at 2*θ* = 26.5° confirmed a graphitic fraction (Fig. 1B). Unpolarized Raman spectra (Fig. 1C) displayed a stronger intensity of the ordered G band at 1585 cm^−1^ (*I*_G_) than the disordered D band at 1397 cm^−1^ (*I*_D_) with *I*_G_/*I*_D_ ≫ 1.26, confirming a high graphitization within the structure.^24^ The C QDs formed agglomerates in dispersion with hydrodynamic diameters from DLS data on the order of 86 ± 7 nm (Fig. 1D). This is in line with reports that C QDs with polar termination groups tend to form hydrogen bonds and agglomerates in the size range of up to 138 nm.^25,26^

The Eu^3+^:CaF_2_ nanocrystals were coated with citrate moieties after synthesis.^23^ TEM revealed spherical nanocrystals with diameters of 9.5 ± 2 nm (Fig. 1E and S1, SI). SAED (Fig. S1, SI) and XRD patterns (Fig. 1F) confirmed that the host structure crystallized as a cubic phase. This host, in addition, displays relatively low phonon energies (approximately 330 cm^−1^), as confirmed from the unpolarized Raman spectra (Fig. 1G). DLS data indicated hydrodynamic diameters of 14 ± 3 nm (Fig. 1H) indicating fully dispersed particles in water.

**Fig. 2:**
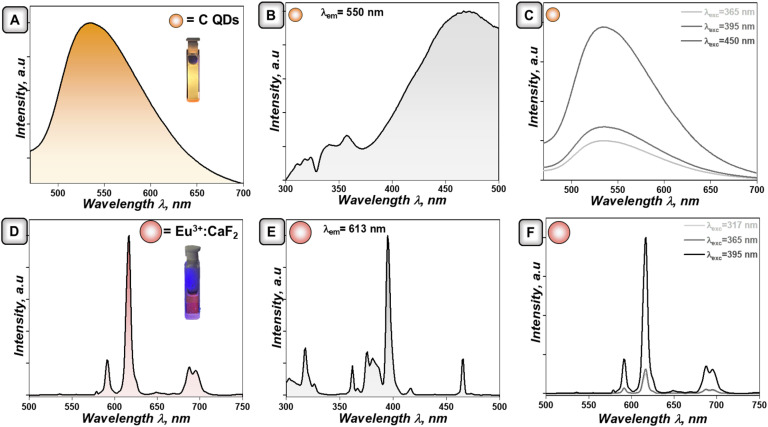
Photoluminescence under 365 nm excitation, excitation spectra and photoluminescence at other excitation wavelengths of the pure particles: (A)–(C) carbon quantum dots and (D)–(F) Eu^3+^:CaF_2_ nanocrystals. Insets are digital images of aqueous dispersions of carbon quantum dots (Fig. 1A) and Eu^3+^:CaF_2_ nanocrystals (Fig. 1D) under 365 nm irradiation. The orange and yellow spherical shapes represent carbon quantum dots and Eu^3+^:CaF_2_ nanocrystals, respectively.

We evaluated the optical properties of the C QDs and Eu^3+^:CaF_2_ nanocrystals separately to identify a single excitation wavelength to trigger emission in both. The C QDs displayed wide absorbance from 350 nm to 580 nm (Fig. S2A, SI). Maxima at 350 nm to 360 nm, and 440 nm to 450 nm (Fig. S2A, SI) are attributed to the n → π* transitions of the functional groups and the π conjugated system within the sp^2^ carbon core,^27^ respectively. The weak shoulder within the range from 510 nm to 580 nm is linked to surface states.^27^ Under 365 nm excitation, the C QDs had their maximum emission at approximately 550 nm (Fig. 2A), visible as bright orange (inset, Fig. 2A). Excitation with monochromatic light between 300 nm and 450 nm indicated the strongest emission at 550 nm for 450 nm excitation (Fig. 2B) with a PLQY of approximately 85 ± 3% (Fig. 2B and C).

The Eu^3+^ doped CaF_2_ nanocrystals had a dominant absorbance band at 395 nm (assigned to the ^7^F_0_ → ^5^L_6_ transition) and lower intensity bands around 365 nm (assigned to the ^5^F_0_ → ^5^G_2_ transition, Fig. S2B, SI).^28^ The dominant emission for 365 nm excitation at 613 nm was assigned to ^5^D_0_ → ^7^F_2_ (Fig. 2D) and appeared red (inset, Fig. 2D). Other detectable emissions were at 580 nm (^5^D_0_ → ^7^F_1_) and at approximately 700 nm (^5^D_0_ → ^7^F_1,2,4_) (Fig. 2D). Multiple weaker peaks appeared within the wavelength range from 410 nm to 540 nm. Other excitation maxima that caused 614 nm emission were at 330 nm, 365 nm, and a dominant 395 nm,^28^ as expected for Eu^3+^ doped materials (Fig. 2E). The emission at 613 nm under 395 nm excitation had a PLQY of approximately 17 ± 2% (Fig. 2E and F).

**Fig. 3:**
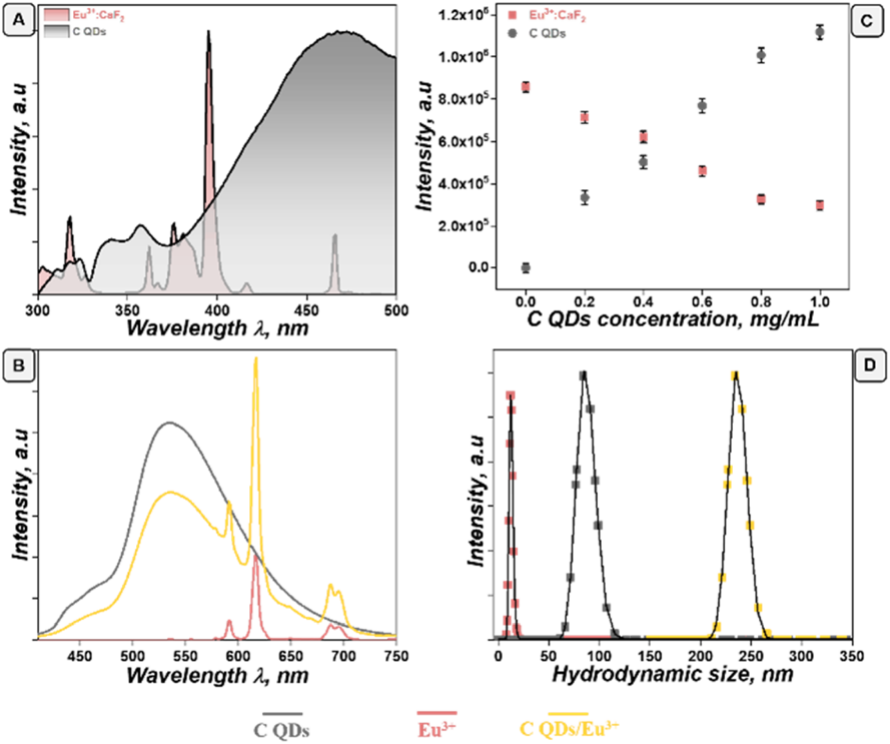
Optical properties and sizes of heteroaggregates. (A) Excitation spectra (550 nm emission for C QDs and 614 nm for europium doped nanocrystals), (B) photoluminescence spectra (under 395 nm excitation), (C) photoluminescence (under 395 nm excitation) as a function of C QD concentration for a constant Eu^3+^:CaF_2_ nanoparticle concentration of 1 mg mL^−1^. (D) Hydrodynamic sizes of carbon quantum dots (in grey), Eu^3+^:CaF_2_ nanocrystals (in red) and their heteroaggregates (in yellow).

In summary, excitation at 395 nm yielded high emission intensities for both particle types (Fig. 3A and B), although the PLQY of the C QDs decreased up to 34 ± 3%. Next, we examined the photostability and photobleaching of the C QDs and Eu^3+^:CaF_2_ nanocrystals. Bleaching causes drift and a reduction in sensitivity and accuracy over time.^29,30^ Both types of particles remained stable under continuous irradiation at 395 nm at a power density of approximately 100 mW cm^−2^ during 24 h. Changes in the emission wavelengths at 550 nm for C QDs and 613 nm for Eu^3+^:CaF_2_ were below the accuracy of the measurement (Fig. S3, SI).

The pure dispersions were now mixed, and we analysed the changes in emission at different particle concentration ratios at 395 nm excitation (Fig. 3A and B) to assess whether coupling led to a deviation from linear superposition. The concentration of C QDs was varied from 0.2 mg mL^−1^ to 1 mg mL^−1^ in 0.2 mg mL^−1^ steps while maintaining the concentration of Eu^3+^:CaF_2_ nanoparticles at 1 mg mL^−1^. Emission at 550 nm (caused by the C QDs) increased with C QD concentration (Fig. 3C). Emission at 613 nm emission (Eu^3+^:CaF_2_ nanoparticles) decreased in the same range at a similar slope. This indicates coupling, most likely by Förster Resonance Energy Transfer (FRET), due to the spectral overlaps. For example, the absorbance band of C QDs in the range of 510 nm to 580 nm matches multiple emissions of Eu^3+^:CaF_2_ (Fig. S4, SI). These changes deviate from pure concentration effects. The inner filter effect (IFE) affects all emission lines proportionally and does not explain the differences between emission bands.

Ratiometric sensing requires strong signals at both recorded wavelengths. We chose a ratio of 0.4 mg mL^−1^ of C QDs and 1 mg mL^−1^ Eu^3+^:CaF_2_; at this ratio, Eu^3+^:CaF_2_ emitted approximately 1.75 times stronger than the C QDs (Fig. 3C). Dynamic light scattering (Fig. 3D) indicates that at this ratio, heteroaggregates formed with hydrodynamic diameters of 237 nm ± 14 (in yellow, Fig. 3D). This increase in apparent particle size from pure dispersions of Eu^3+^:CaF_2_ nanoparticles with 14 ± 3 nm (in red, Fig. 3D) and pure C QDs with 86 ± 7 nm (in grey, Fig. 3D) indicates that assembly took place. The pure Eu^3+^:CaF_2_ nanoparticles had negative zeta potentials of −32 ± 1.7 mV, while pure C QDs were neutral (0.5 ± 0.2 mV). Upon mixing, heteroaggregates formed with a potential of −22 ± 3.1 mV.

The heteroaggregates were tested as sensor probes for temperature and pH in aqueous media with ratiometric readout. Readout of two independent wavelengths reduces the effects of the concentration of the fluorescent unit, therefore reducing measurement errors.^4,16^ We explored how the intensities of the two dominant bands of the C QDs (at 550 nm) and Eu^3+^:CaF_2_ materials (at 613 nm) change as a function of temperature and pH in aqueous media in the physiological range that is relevant to many biomedical applications.^4,16^

**Fig. 4:**
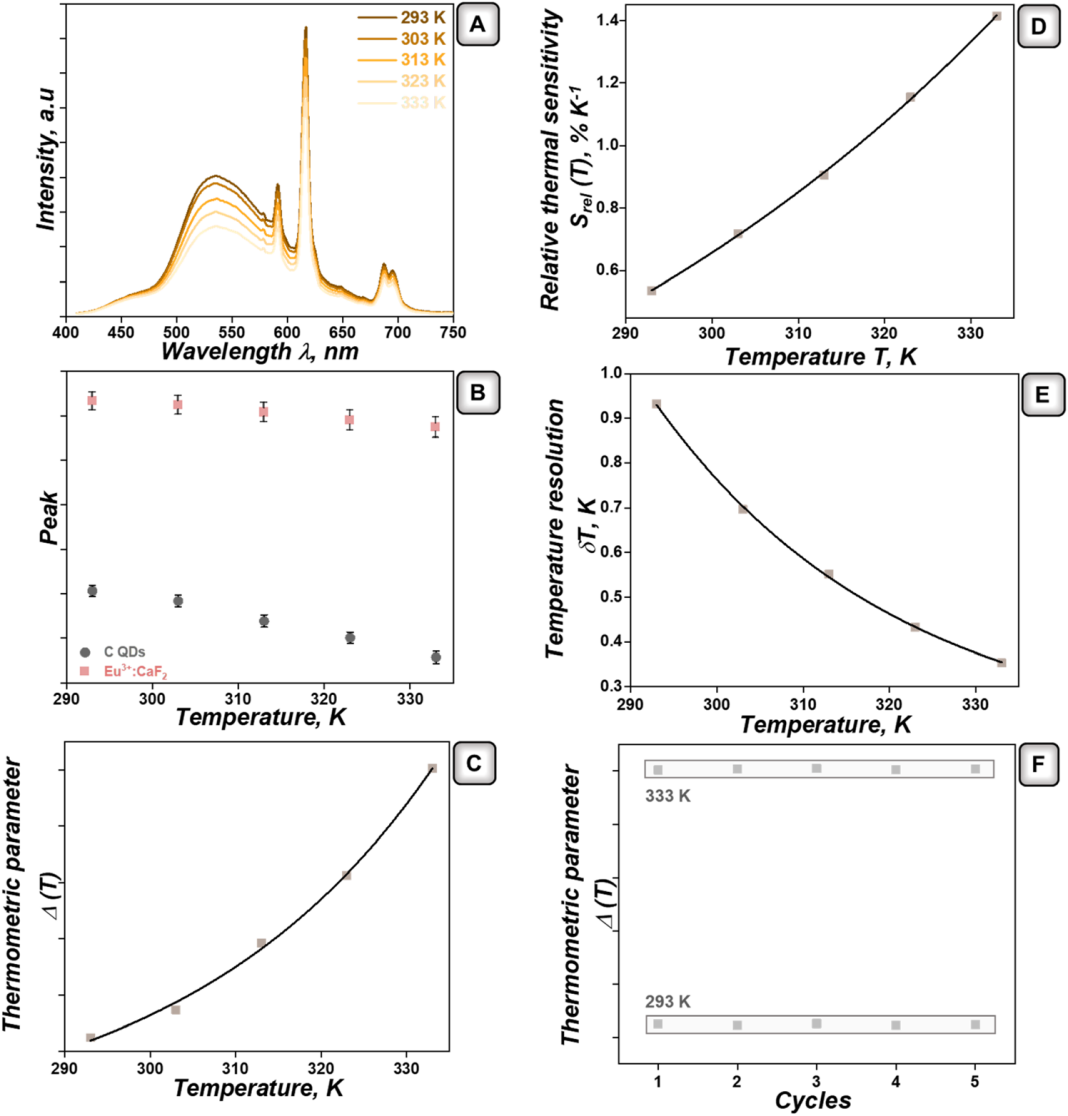
Heteroaggregates of C QDs and Eu^3+^:CaF_2_ nanocrystals as luminescent thermometers. (A) Temperature dependence of the photoluminescence. (B) Evolution of the peak of the C QDs (550 nm, in grey) and Eu^3+^:CaF_2_ (613 nm, in red), (C) thermometric parameter, (D) relative thermal sensitivity, (E) temperature resolution and (F) repeatability, as a function of temperature.

The emission of Eu^3+^:CaF_2_ nanocrystals at 613 nm alone decreased by 17% when heating from 293 K to 333 K, while that of pure C QDs decreased by 33% in the same range (Fig. S5, SI). The photoluminescence of heteroaggregates containing C QDs and Eu^3+^:CaF_2_ at 550 nm decreased by approximately 37%, while that at 613 nm by 8% of its initial value (Fig. 4A). The drop at 613 nm was nearly linear with temperature (red symbols, Fig. 4B), the decrease at 550 nm was exponential (grey symbols, Fig. 4B).

The thermometric performance of the heteroaggregates was evaluated based on the calculation of the thermometric parameter (Δ(*T*)), the relative thermal sensitivity (*S*_rel_(*T*)), the repeatability (*R*), and the temperature resolution (*δT*). These are figures of merit established for luminescent thermometers.^4,16^ The thermometric parameter Δ(*T*) links the intensity ratio among two emission bands with temperature.^4,16^ The ratio Δ(*T*) followed an exponential function:1
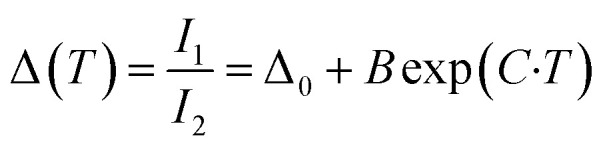
where *I*_1_ and *I*_2_ are the intensities at 550 nm and 613 nm, respectively, while Δ_0_, *B* and *C* are fitting constants with the following values:

2 Δ(*T*) = 1.70 + 1.65 × 10^−5^ exp(0.0338·*T*)

The relative thermal sensitivity *S*_rel_(*T*) quantifies the change of Δ(*T*) with temperature and is commonly used to compare the performance of luminescent thermometers, independent of the nature of the fluorescent units or the acquisition setup.^4,16^ We calculated it as:^4,16^3
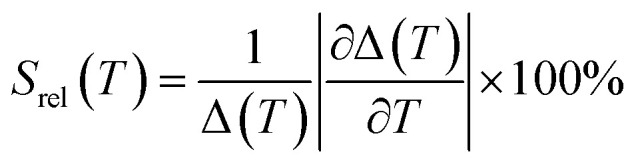


using the fits of eqn (1) above:4
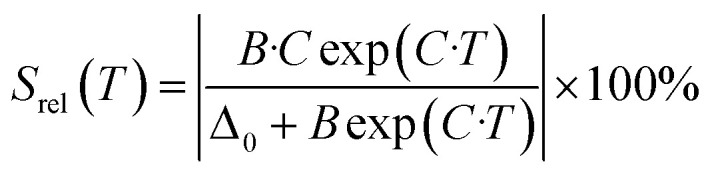


The temperature-dependent *S*_rel_(*T*) were between 0.53 K^−1^ (at 293 K) and 1.41 K^−1^ (at 333 K) (Fig. 4D).

We used *S*_rel_(*T*) as a figure of merit for the thermometric performance and compared its values to the published state of the art.^4,16^ Most studies of the temperature dependence of the photoluminescence of C QDs^31^ report a single wavelength rather than intensity ratios.^32^ We only consider reports that used more than one emission.^12,31,33^ The maximum *S*_rel_(*T*) reported for the range 333 K to 343 K were in the range of 1.21% K^−1^ (at 333 K)^12^ to 1.8% K^−1^ (at 343 K).^31^ Our heteroaggregates match these values and exceed them at 333 K (1.41% K^−1^). Larger values have been reported for C QDs emitting at the blue wavelength combined with micron-scale Eu^3+^ doped KMgF_3_ particles that had *S*_rel_(*T*) of 2% K^−1^ at 300 K,^22^ but their optical scattering limits applicability.

The temperature resolution *δT* expresses the minimal temperature change that a luminescent thermometer can detect and is defined as:^4,16^5
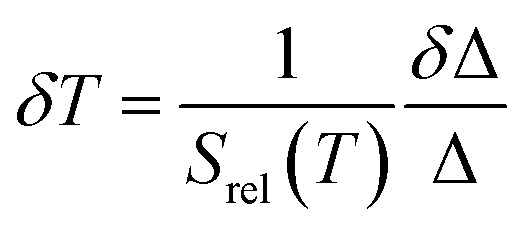


with 
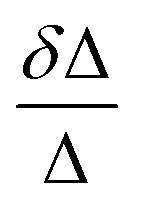
 being the degree of uncertainty in determining the thermometric parameter Δ. It reached its smallest value (*i.e.*, best thermometric performance) at 333 K, where our thermometer can resolve 0.35 K changes in temperature (Fig. 4E). We did not find any values of *δT* reported in the open literature for C QD based thermometers.

Finally, we analysed the repeatability *R* during 5 heating/cooling cycles (293 K to 333 K). This parameter quantifies the ability of a thermometer to provide the same thermal readings (either *S*_rel_ or Δ) at the same temperature and is given as:^4,16^6
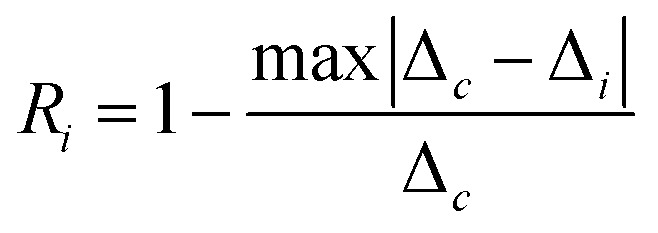


We found a repeatability of 99 ± 3% during 5 cycles, demonstrating a reliable performance of these types of thermometers. This value fits within the range (95% to 99%) reported from the state of the art.^22,31,33^

**Fig. 5:**
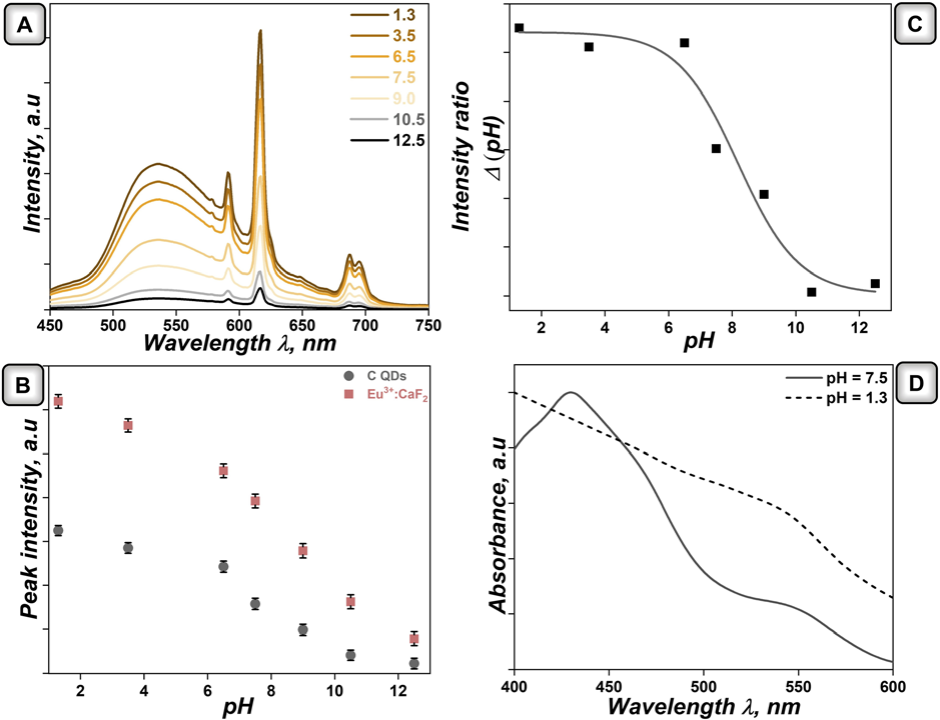
Heteroaggregates of C QDs and Eu^3+^:CaF_2_ nanocrystals as luminescent pH sensors. (A) pH dependence of the photoluminescence and (B) evolution of the peak of the C QDs (550 nm, in yellow) and Eu^3+^:CaF_2_ (613 nm, in red) as a function of pH. (C) Calibration curve as a function of pH. (D) Absorbance of C QDs as a function of the pH (dashed line: acid pH 1.3, and solid line: neutral pH 7.5).

We tested the optical response of the heteroaggregates to changes in pH within phosphate buffer saline (PBS) solutions (Fig. 5A). Emission at both 550 nm and 613 nm decreased in intensity with pH in the entire range (Fig. 5A and B), more strongly at 550 nm (by 92%) than at 613 nm (by 74%). The emissions of pure C QDs and Eu^3+^:CaF_2_ nanocrystals reduced by 62% and 47%, respectively (Fig. S6, SI). The decreases were linear with a slope of −0.191 until pH 6.5, then remained linear but with a slope of −0.389 until pH 10.5, and became less sensitive above, particularly at 550 nm (Fig. 5C).

We used the calculated Δ(pH), as the intensity ratio between the 550 nm and 613 nm emissions, to construct a calibration curve of the heteroaggregates. The curve was sigmoidal with *R*^2^ = 0.963, displaying an exponential change with pH (Fig. 5C). To understand these changes, we monitored the absorbance of the pure C QDs. For that, we recorded the absorbance of the C QDs at acidic (1.3) and neutral (7.5) pH. At neutral pH, the absorbance of the C QDs was similar to that after synthesis (Fig. S2, SI) with a clear band at approximately 450 nm (solid lines, Fig. 5D). This band completely disappeared for acidic pH (dashed line, Fig. 5D), which explains the significant initial drop of the intensity of the photoluminescence of the C QDs. We recorded the hydrodynamic sizes of the heteroaggregates at pH 1.3 and pH 7.5 and only found statistically irrelevant changes (Fig. S7, SI).

We calculated the sensitivity and resolution for pH following previous approaches.^34,35^ The relative pH sensitivity was calculated as:^34,35^7
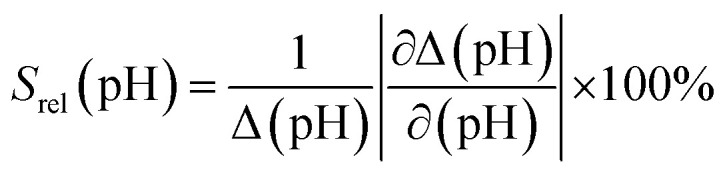


and the resolution as:^34,35^8
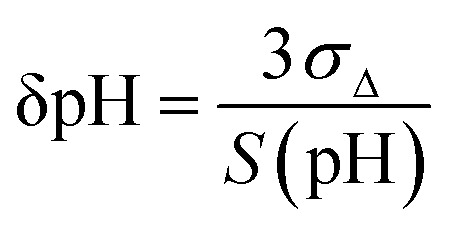
where Δ (pH) is the peak ratio among the 550 nm and 613 nm emissions as a function of pH, σ_Δ_ the standard deviation of the peak ratio and *S*(pH) the absolute sensitivity as 
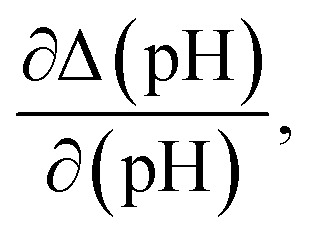
 respectively.^34,35^ Our highest *S*_rel_(pH) was 27% per unit pH, detected within the steepest drop of intensity peaks at pH from 6.5 to 9.0. The resolution *δ*pH was within 0.15 pH units at this pH range.

Overall, our ratiometric sensing approach tackles the current limitations of temperature and pH sensor probes that operate mainly on single wavelength emissions. By creating heteroaggregates, we improved the sensing performance of pure materials. For thermal readout, the pure materials displayed maximum changes until 33%, while the changes in heteroaggregates reached 37%. Changes with pH increased from 62% for pure materials up to 92% for heteroaggregates. This enhancement is caused by the coupling of particles and FRET in the heteroaggregates.

## Conclusions

We assembled carbon quantum dots and Eu^3+^ doped CaF_2_ nanocrystals. The resulting heteroaggregates exhibited electromagnetic coupling between the constituent nanoparticles. We demonstrate their use as temperature and pH sensors for ratiometric readout that enables direct recovery of absolute measurement values. Signal intensities and attainable measurement performance increased beyond the performance of the constituent particles by the coupling phosphors.

The heteroaggregates formed here can be integrated into polymer matrices such as cellulose,^36^ or polylactic acid derivatives.^37^ This would yield composite sensor materials that are suitable for the integration of sensing into the structure, *e.g.*, of soft robots. Degradable polymer matrices, such as polylactic acid,^6^ enable the design of probes that can remain in the environment.

## Author contributions

Albenc Nexha conducted the experiments and wrote the original draft of the manuscript. Tobias Kraus revised the manuscript, led the supervision and funding acquisition.

## Conflicts of interest

There are no conflicts to declare.

## Supplementary Material

NA-OLF-D6NA00343E-s001

## Data Availability

The data that support the findings of this study will be made available upon request from the corresponding authors. Supplementary information (SI) is available. See DOI: https://doi.org/10.1039/d6na00343e.
