# Supplementary material for: Ratiometric multisensing with heteroaggregates of aqueous carbon quantum dots and rare earth doped nanocrystals
Source: Nanoscale Adv. 2026 Jul 13. Online ahead of print. doi: 10.1039/d6na00343e (PMC13386136; doi:10.1039/d6na00343e)
Supplement: NA-OLF-D6NA00343E-s001 [file NA-OLF-D6NA00343E-s001.pdf]

Supplementary Information (SI) for Nanoscale Advances.
This journal is © The Royal Society of Chemistry 2026

SUPPORTING INFORMATION

Ratiometric multisensing with heteroaggregates of aqueous carbon quantum dots and rare earth doped nanocrystals

Albenc Nexha[1] **and Tobias Kraus**[1,2*]
[1]*INM-Leibniz Institute for New Materials, Campus D2 2, 66123 Saarbrücken, Germany*
[2]*Saarland University, Colloid and Interface Chemistry, 66123 Saarbrücken, Germany*
*tobias.kraus@leibniz-inm.de

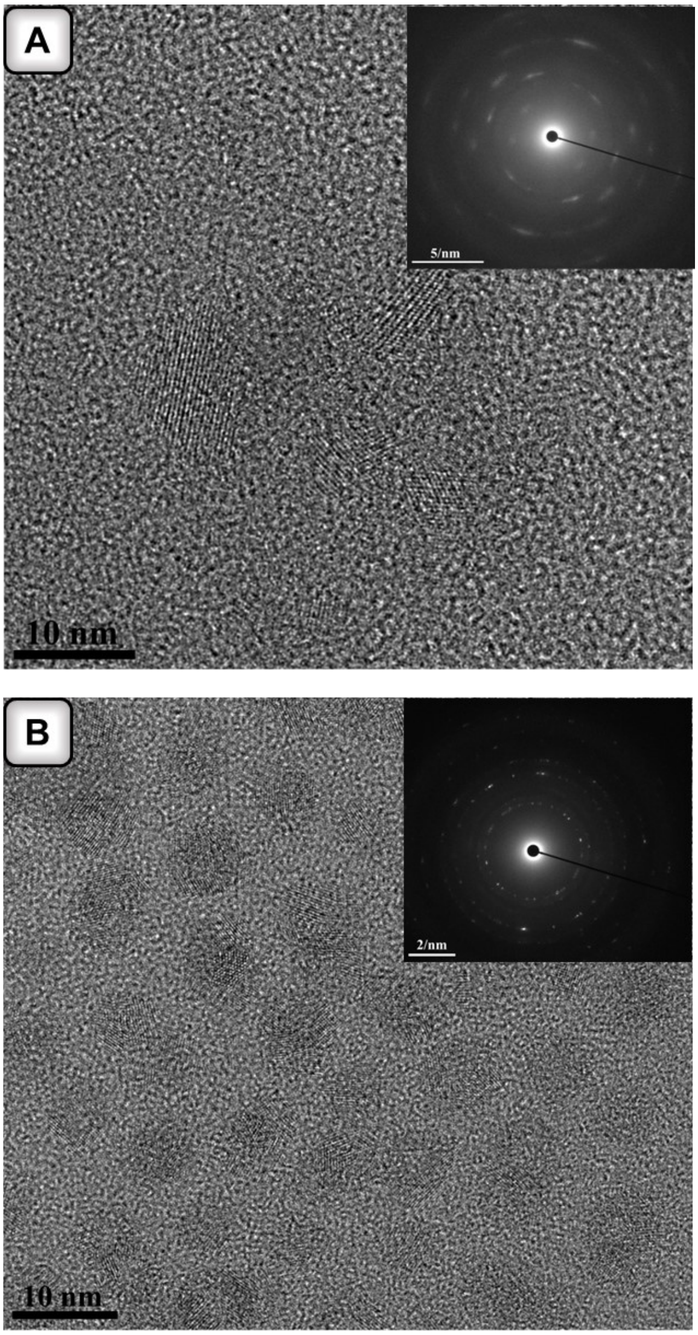

Figure S1. High resolution transmission electron images and selected area electron diffraction (insets) of: (A) carbon and (B) Eu^{3+} doped CaF_2 nanocrystals.

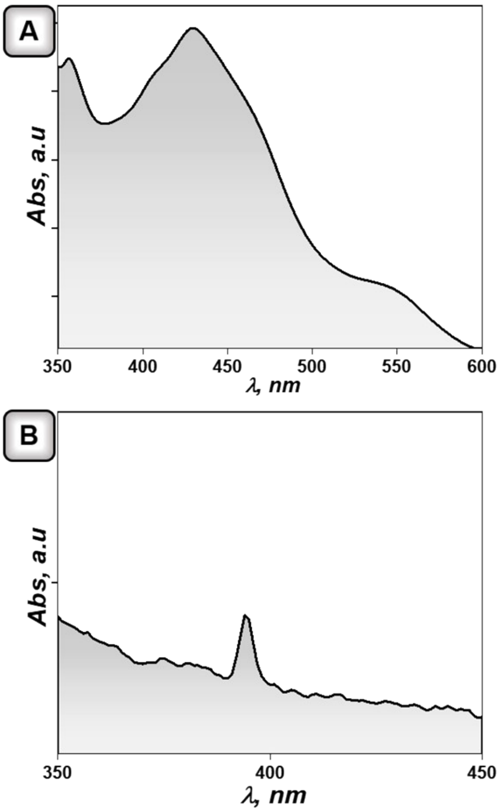

Figure S2. Absorbance spectra of (A) carbon quantum dots and (B) Eu^{3+} doped CaF_2 nanocrystals in water.

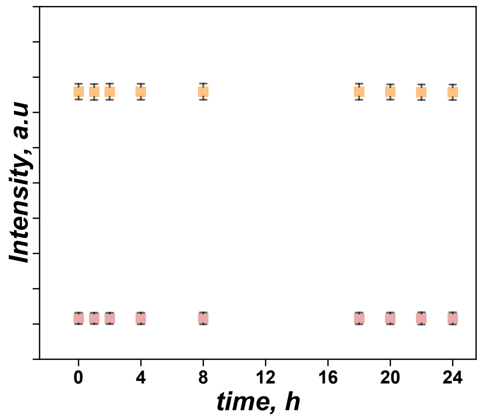

Figure S3. Photostability of carbon quantum dots (in orange) and europium doped nanocrystals under continous 365 nm irradiation for 24 hours.

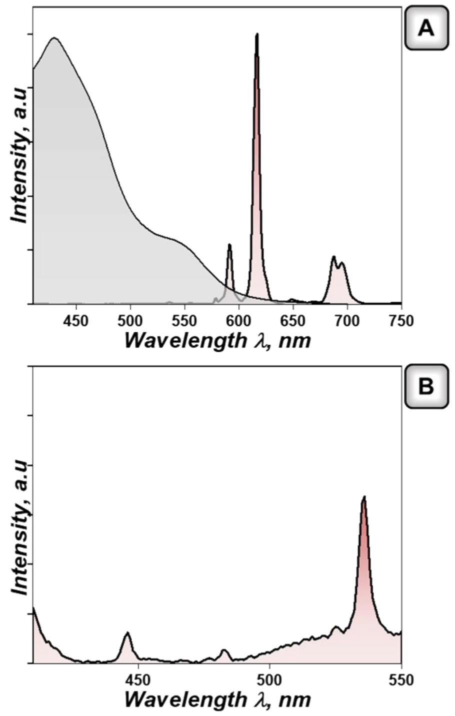

Figure S4. (A) Absorbance of carbon quantum dots (in grey) and photoluminescence of Eu^{3+} doped nanocrystals (in red), (B) zoomed in graph of photoluminescence of Eu^{3+} doped nanocrystals within 410 nm to 550 nm.

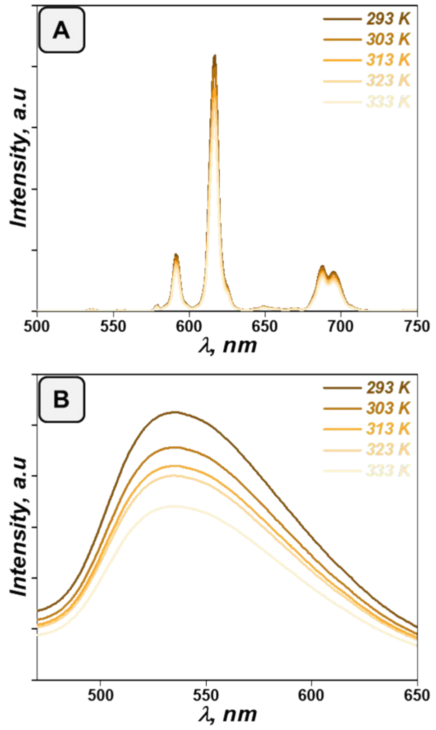

Figure S5. Temperature dependence of the photoluminescence of pure (A) Eu^{3+} doped nanocrystals and (B) carbon quantum dots.

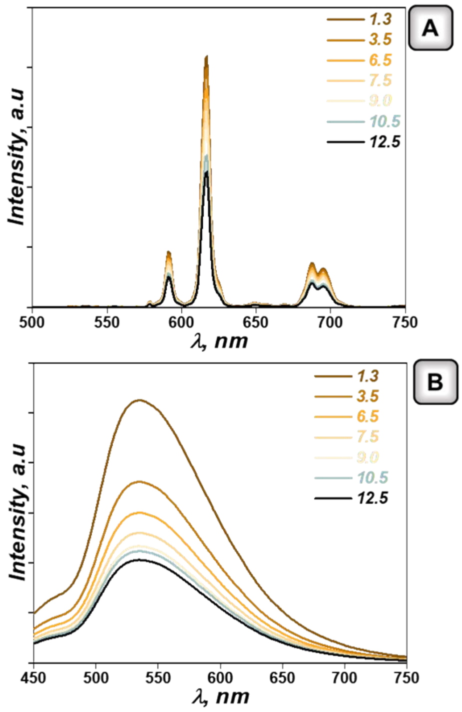

Figure S6. pH dependence of the photoluminescence of pure (A) Eu^{3+} doped nanocrystals and (B) carbon quantum dots.

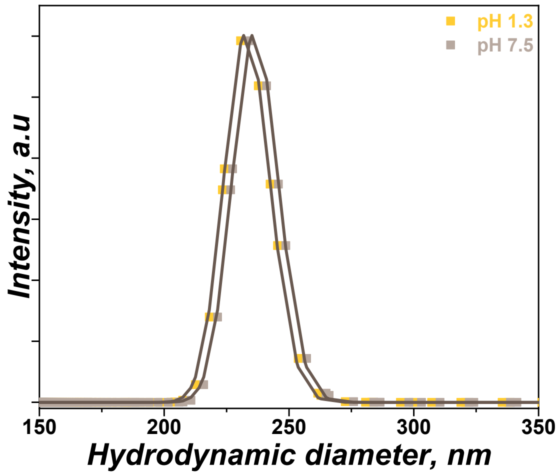

Figure S7. Hydrodynamic size of the heteroagglomerates at pH 1.3 and pH 7.5.
